# The Homœopathic Therapeutics of Hemorrhoids

**Published:** 1892-06

**Authors:** 


THE HOMŒOPATHIC THERAPEUTICS OF HEMORRHOIDS. By Wm. Jefferson Guernsey, M. D. (Second Edition). Philadelphia: Bœricke & Tafel, 1011 Arch Street, 1892. Price, $1.00, net.

The first edition of this book was published several years ago, as a supplement to THE HOMŒOPATHIC PHYSICIAN. It consisted of a repertory and a short *resumé* of the materia medica of hemorrhoids, making in all 25 pages. The present volume is a much larger work of 140 pages, in which the larger portion is devoted to materia medica and followed by the Repertory at the end, producing a book that somewhat resembles the first edition of *Bell on Diarrhœa.*

The materia medica is excellent, the symptoms under each remedy being divided into "Subjective," "Objective," "Aggravation," "Amelioration," and "Concomitant." The Repertory is divided into chapters having similar headings. The Repertory in this new edition does not seem to us as well arranged as in the first edition. However, it is a very good book, and will be warmly welcomed by every Hahnemannian. W. M. J.

